# Regional Lymph Node Metastasis and Axillary Surgery of Microinvasive Breast Cancer: A Population-Based Study

**DOI:** 10.3390/diagnostics12051049

**Published:** 2022-04-21

**Authors:** Jiamei Chen, Bo Luo, Mengting Gao, Gaoke Cai, Xixi Luo, Yutian Zhang-Cai, Shaobo Ke, Yongshun Chen

**Affiliations:** 1 Center of Oncology, Renmin Hospital of Wuhan University, Wuhan 430060, China; chenjiamei@whu.edu.cn (J.C.); 2014302180109@whu.edu.cn (G.C.); xixiluo@whu.edu.cn (X.L.); zhangcaiyt1995@whu.edu.cn (Y.Z.-C.); 2 Department of Pathology, The Central Hospital of Wuhan, Tongji Medical College, Huazhong University of Science and Technology, Wuhan 430014, China; luobo198705051@126.com; 3 Center of Information, Renmin Hospital of Wuhan University, Wuhan 430060, China; xfbtting@163.com

**Keywords:** microinvasive breast cancer, lymph node metastasis, axillary surgery, axillary lymph node dissection, Surveillance Epidemiology and End Results

## Abstract

Microinvasive breast cancer (MBC for short) is a rare entity with the decision of axillary surgery under debate in clinical practice. We aimed to unravel the lymph node metastasis (LNM) rate, axillary surgery, and prognosis of MBC based on 11,692 patients derived from the Surveillance Epidemiology and End Results (SEER) database between 2003 and 2015. In this retrospective study, 19.5% (2276/11,692) of patients received axillary lymph node dissection (ALND), 80.5% (9416/11,692) received non-ALND. In the total cohort, 10-year breast cancer-specific survival (BCSS) was 96.3%, and the LNM rate was 6.4% (754/11,692). Multivariate analyses showed that LNM had the strongest predictive weight (N3, HR 14.200, 95% CI 7.933–25.417; N2, HR 12.945, 95% CI 7.725–21.694; N1, HR 3.05, 95% CI 2.246–4.140, all *p* < 0.001). Kaplan–Meier analyses showed that ALND did not confer a survival benefit on 10-year BCS in patients with N0 (94.7% vs. 97.1%, *p* < 0.001) and in patients with 1–2 positive nodes (92.1% vs. 89.5%, *p* = 0.355), respectively, when compared to non-ALND. Our study demonstrated that the vast majority of MBC have a low LNM rate and excellent prognosis; patients with LNM showed poor prognosis. Assessment of lymph node status is necessary, and non-ALND surgery is required and sufficient for MBC with 0–2 positive nodes.

## 1. Introduction

Breast cancer (BC) is a highly heterogeneous disease with various pathological and molecular subtypes [1]. Microinvasive breast cancer (MBC for short) is a rare subtype of BC with clinicopathological characteristics needing further elucidation [2]. It is defined as ductal carcinoma in situ (DCIS) with one or more foci of tumor cells extending beyond the basement membrane, and no focus is larger than 1 mm in max diameter [3]. Though it has been classified as pT1mi that belongs to T1 in the American Joint Committee on Cancer (AJCC) staging system, the decision of axillary surgery and adjuvant therapy for MBC is under debate in clinical practice [2,4,5]. Whether MBC should be managed similarly to pure DCIS or invasive BC remains an open question, particularly for axillary staging. Because regional lymph node status is a stronger predictor of adverse outcomes and critically impacts subsequent treatment decisions.

The latest meta-analysis showed that MBC with the lymph node macrometastases rate of 2% had similar survival to DCIS and suggested that routine axillary staging may be an unnecessary intervention that does not change the management of MBC [6]. Sentinel lymph node biopsy (SLNB), a minimal invasion method that has comparable accuracy with axillary lymph node dissection (ALND), has become the mainstay in axillary staging for early breast cancer [7]. Magnoni et al. [8] reported that MBC had a favorable long-term survival and suggested that even SLNB in MBC may not be applicable. However, some researchers underline the impact of microinvasion on survival and suggest that MBC may have more aggressive pathologic features than DCIS and needs axillary staging [4,9,10], especially for younger patients with SLN metastases [11].

In terms of the inconsistent findings, our retrospective study supplements the existing research on regional lymph node metastasis (LNM) incidence, axillary surgery, and prognosis of MBC based on 11,692 patients pooled from the Surveillance Epidemiology and End Results (SEER) dataset.

## 2. Patients and Methods

### 2.1. Database and Patients

This retrospective study was based on the 18 Registries Incidence-SEER Database (November 2020 submission) of the National Cancer Institute, which collects incidence, clinicopathological features, treatment, and outcomes data from 18 cancer registries in the United States. This study was conducted in accordance with the guidelines of the Helsinki Declaration. Patients were included when {Site and Morphology Primary Site-labeled = C50.0-C50.9 breast} AND “{Derived AJCC, 7th ed (2010–2015) T = T1mi} OR {Derived AJCC, 6th ed (2004–2015) T = T1mi}”. Patients were eliminated when they contained the following situation: non-epithelial tumors, diagnosed < 18 years, AJCC IV stage, with the number of resected or positive nodes unknown, without node pathological examination, or with the survival months of zero.

### 2.2. Data Extraction

Relevant variables of patients were derived via SEER*Stat v8.3.9. Demographic characteristics include patient ID, age, sex, years of diagnosis, race, diagnostic confirmation, tumor history, and sequence number of BC when suffering from multiple tumors. Clinicopathological features were primary tumor site, histological type, grade, estrogen receptor (ER), progesterone receptor (PR), human epidermal growth factor receptor 2 (HER2) status, molecular subtype, AJCC Tumor-lymph Node-Metastasis (TNM) stage, number of examined lymph nodes, and number of positive nodes. The grade was classified into 1 (grade I, well-differentiated), 2 (grade II, moderately-differentiated), and 3 (grade III/IV, poorly-differentiated/undifferentiated). Histological types were classified into four groups, favorable type (adenoid cystic, tubular, encapsulated papillary, mucinous, and cribriform carcinoma), non-special type (NST) and others (ductal/lobular NST, intracystic, medullary, and apocrine carcinoma), poor type (micropapillary, clear cell, signet ring cell, and metaplastic carcinoma), and unspecified carcinoma.

As the SEER dataset does not document axillary surgery type, patients were grouped into the ALND subset (six or more nodes examined) and non-ALND subset (1–5 nodes examined) based on the AJCC definition of ALND [12]. Nodes with macrometastases and micrometastases (N1mi) were defined as N+, while negative nodes, nodes with isolated tumor cells (ITCs), N0 (i+), and N0 (mol+) were defined as N0. Breast cancer and non-breast cancer cause of death, vital status, and survival months were derived. Breast cancer-specific survival (BCSS) was determined from the date of diagnosis to the date of BC-associated death.

### 2.3. Statistical Analysis

Data were first characterized by descriptive statistics, then presented as a percentage for categorical variables and median (range) for continuous variables. Differences between groups were compared using the Chi-square test with Bonferroni correction. Survival outcomes were compared by Kaplan–Meier with the log-rank test. Multivariate Cox proportional hazard regression analysis was performed to identify prognostic predictors for 10-year BCSS. Propensity score matching (PSM) was used to create matched pairs by year of diagnosis to avoid potential inference from imbalanced data. All statistical analyses were processed using the SPSS 26.0 (SPSS Inc., Chicago, IL, USA) and the packages in R version 4.1.0 (R Foundation for Statistical Computing, Vienna, Austria). Two sides *p* < 0.05 was considered statistically significant.

## 3. Results

**Figure 1:**
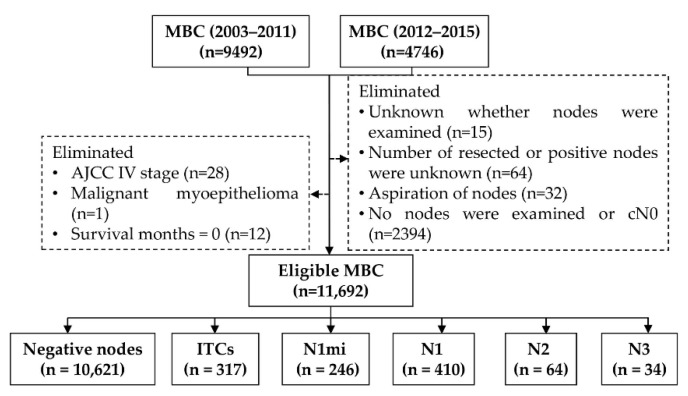
The flow chart of patient inclusion and exclusion. AJCC: American Joint Committee on Cancer, ITCs: isolated tumor cells, MBC: microinvasive breast cancer, N1: 1–3 positive nodes, N2: 4–9 positive nodes, N3: ≥10 positive nodes, N1mi: micrometastases nodes.

**Figure 2:**
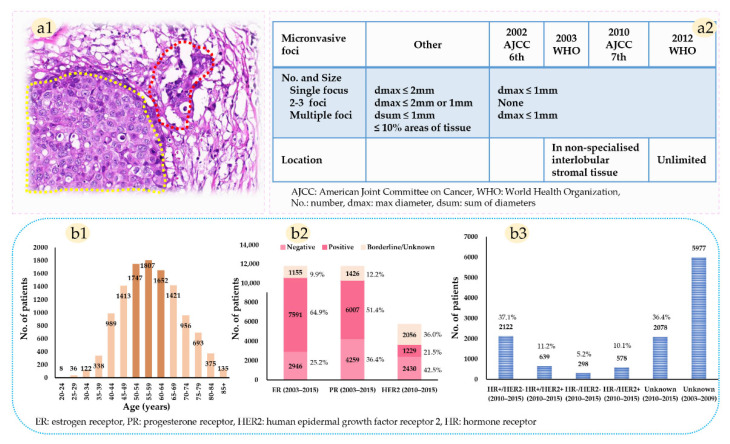
The diagnostic criterion of microinvasive breast cancer (MBC) and major clinicopathological characteristics of the eligible population. (**a1**) MBC is defined as ductal carcinoma in situ (yellow dash line) with one or more foci of tumor cells (red dash line) extending beyond the basement membrane. No focus is larger than 1 mm in max diameter. (**a2**) Evolution of the diagnostic criterion for microinvasion. A unified measurement standard for microinvasion was first established in the AJCC 6th edition manual. Subsequently, the 2003 WHO classification of breast tumors recommended that the tumor cells in non-specialized interlobular, not cells in interlobular stromal tissue, were regarded as microinvasions. The constraint for the location of tumor cells did not change until the 2012 WHO classification of tumors. (**b1**) Age distribution of the eligible population. (**b2**) ER, PR, HER2 distribution of the eligible population, and the HER2 status was not included in the SEER data until 2010. (**b3**) Molecular subtypes distribution, and the subtype was not available until 2010 accordingly.

Finally, 11,692 patients with primary MBC diagnosed between 2003 and 2015 were included (Figure 1). Patients diagnosed before 2003 were excluded because the diagnostic criterion for MBC is an evolving process that is constantly being refined until 2003 (Figure 2(a1,a2)).

### 3.1. Demographic and Clinicopathological Characteristics

The clinicopathological characteristics of the eligible population are detailed in Table 1. About 56.7% (6627/11,692) of patients were in the age category of 50–69 years (Figure 2(b1)), and the vast majority of histological type was NST and others (95.3%). Except for borderline/unknown status, there were 25.2% negative ER (ER−), 64.9% positive ER (ER+), 42.5% negative (HER2−), and 21.5% positive HER2 (HER2+) (Figure 2(b2)). For patients diagnosed in 2010 or later, 48.3% were luminal subtype (HR+/HER2− or HR+/HER2+), 10.1% were HER2 enriched-type (HR-/HER2+), and 5.2% were triple-negative (TNBC, HR-/HER2−) (Figure 2(b3)).

As a discrepancy exists between before and after 2012 on the definition of microinvasion location (Figure 2(a2)), a comparison of the baseline characteristics was conducted. Compared to patients diagnosed in 2012–2015, patients in 2003–2011 had a higher proportion of LNM, grade 3, AJCC III stage, ALND, and chemotherapy. However, there were no significant differences in histological type, molecular subtype, primary site surgery, and radiotherapy (Table 1).

**Table 1:** Baseline demographic and clinicopathological characteristics of 11,692 microinvasive breast cancer.

Characteristics	Total n (%)	2003–2011 n (%) (n = 7827)	2012–2015 n (%) (n = 3865)	*p*-Value
Age, years				<0.001
20–49	2906 (24.8)	2013 (25.7) ^a^	893 (23.1) ^b^	
50–69	6627 (56.7)	4309 (55.1) ^a^	2318 (60.0) ^b^	
≥70	2159 (18.5)	1505 (19.2) ^a^	654 (16.9) ^b^	
Race				<0.001
White	8948 (76.5)	6095 (77.9) ^a^	2853 (73.8) ^b^	
Black	1308 (11.2)	862 (11.0) ^a^	446 (11.5) ^a^	
Other ^①^/Unknown	1436 (12.3)	870 (11.1) ^a^	566 (14.7) ^b^	
Histological type				0.523
Favorable type ^②^	314 (2.7)	221 (2.8)	93 (2.4)	
NST and others ^③^	11,142 (95.3)	7446 (95.1)	3696 (95.6)	
Poor type ^④^	33 (0.3)	24 (0.3)	9 (0.2)	
Unspecified carcinoma	203 (1.7)	136 (1.7)	67 (1.7)	
Grade				<0.001
1	1923 (16.4)	1247 (15.9) ^a^	676 (17.5) ^b^	
2	3100 (26.5)	2019 (25.8) ^a^	1081 (28.0) ^b^	
3	2796 (23.9)	2063 (26.4) ^a^	733 (19.0) ^b^	
Unknown	3873 (33.1)	2498 (31.9) ^a^	1375 (35.6) ^b^	
ER				<0.001
Negative	2946 (25.2)	2044 (26.1) ^a^	902 (23.3) ^b^	
Positive	7591 (64.9)	4788 (61.2) ^a^	2803 (72.5) ^b^	
Borderline/Unknown	1155 (9.9)	995 (12.7) ^a^	160 (4.1) ^b^	
PR				<0.001
Negative	4259 (36.4)	2862 (36.6) ^a^	1397 (36.1) ^a^	
Positive	6007 (51.4)	3753 (47.9) ^a^	2254 (58.3) ^b^	
Borderline/Unknown	1426 (12.2)	1212 (15.5) ^a^	214 (5.5) ^b^	
HER2 (from 2010)				0.018
Negative	2430 (42.5)	766 (41.4) ^a^	1664 (43.0) ^a^	
Positive	1229 (21.5)	372 (20.1) ^a^	857 (22.2) ^a^	
Borderline/Unknown	2056 (36.0)	712 (38.5) ^a^	1344 (34.8) ^b^	
Molecular subtype (from 2010)				0.044
Luminal	2761 (48.3)	853 (46.2)	1908 (49.4)	
Triple-negative	298 (5.2)	97 (5.2)	201 (5.2)	
HER2 enriched	578 (10.1)	180 (9.7)	398 (10.3)	
Unknown	2078 (36.4)	720 (38.9)	1358 (35.1)	
N stage				<0.001
N0	10,938 (93.6)	7265 (92.8) ^a^	3673 (95.0) ^b^	
N1	656 (5.6)	478 (6.1) ^a^	178 (4.6) ^b^	
N2	64 (0.5)	55 (0.7) ^a^	9 (0.2) ^b^	
N3	34 (0.3)	29 (0.4) ^a^	5 (0.1) ^b^	
AJCC stage				<0.001
I	11,182 (95.7)	7445 (95.1) ^a^	3737 (96.7) ^b^	
II	413 (3.5)	299 (3.8) ^a^	114 (2.9) ^b^	
III	97 (0.8)	83 (1.1) ^a^	14 (0.4) ^b^	
Primary site				<0.001
Inner	1874 (16.0)	1189 (15.2) ^a^	685 (17.7) ^b^	
Outer/Axillary tail	4928 (42.1)	3267 (41.7) ^a^	1661 (43.0) ^a^	
Other	4890 (41.8)	3371 (43.1) ^a^	1519 (39.3) ^b^	
Sequence No. of breast cancer				<0.001
One primary only	8059 (68.9)	5231 (66.8) ^a^	2828 (73.2) ^b^	
1st of multi-tumors	1483 (12.9)	1160 (14.8) ^a^	323 (8.4) ^b^	
≥ 2nd of multi-tumors	2150 (18.4)	1436 (18.3) ^a^	714 (18.5) ^a^	
Primary site surgery				0.599
No/unknown	15 (0.1)	11 (0.1)	4 (0.1)	
Yes	11,677 (99.9)	7816 (99.9)	3861 (99.9)	
Axillary surgery				<0.001
non-ALND (1–5 nodes)	9416 (80.5)	6007 (76.7)	3409 (88.2)	
ALND (≥6 nodes)	2276 (19.5)	1820 (23.3)	456 (11.8)	
Chemotherapy				<0.001
No/unknown	10,687 (91.4)	7099 (90.7)	3588 (92.8)	
Yes	1005 (8.6)	728 (9.3)	277 (7.2)	
Radiotherapy				0.020
Yes	5098 (43.6)	3401 (43.5) ^a^	1697 (43.9) ^a^	
No/unknown	6511 (55.7)	4382 (56.0) ^a^	2129 (55.1) ^a^	
Refused	83 (0.7)	44 (0.6) ^a^	39 (1.0) ^b^	

Notes: ① Other races are Asian or Pacific Islander and American Indian/Alaska Native. ② Favorable types included adenoid cystic, tubular, encapsulated papillary, mucinous, and cribriform carcinoma. ③ NST and others include ductal and lobular carcinoma non-special type (NST), intracystic, medullary, and apocrine carcinoma. ④ Poor type includes micropapillary, clear cell, signet ring cell, and metaplastic carcinoma. a, b: Different marks a and b indicate that pairwise comparisons are statistically significant between subgroups. The same marks indicate not statistically significant, such as a and a.

### 3.2. Incidence of LNM

Approximately 99.9% patients received primary tumor excision, followed with ALND (19.5%, 2276/11,692), or non-ALND (80.5%, 9416/11,692), respectively (Table 1). LNM occurred in 6.4% (754/11,692) patients including 4.3% (508/11,692) macrometastases and 2.1% (246/11,692) N1mi. In non-ALND subgroup, there were 1.3% (127/9416) macrometastases, 1.6% (148/9416) N1mi, and 2.8% (261/9416) ITCs.

### 3.3. Survival Outcomes and Prognostic Predictors for BCSS

With a median follow-up of 96 months (range, 1–191 months), 2.9% (334/11,692) breast cancer-associated deaths were reported, including 253 (2.3%) in N0 subgroup and 81 (10.7%) in N+ subgroup, 10-year BCSS was 96.3% in total cohort.

**Figure 3:**
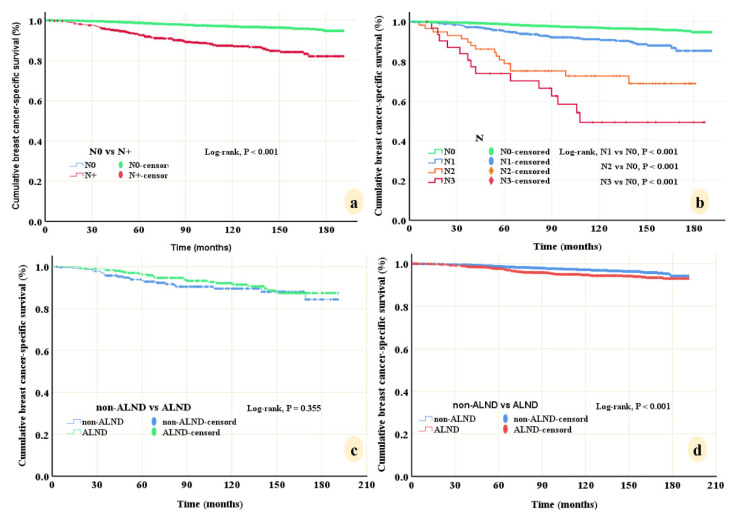
Prognostic value of lymph node metastases of breast cancer-specific survival (BCSS), and survival benefit of axillary lymph node dissection (ALND) for microinvasive breast cancer. (**a**) N+ subgroup had lower 10-year BCSS than N0 subgroup (87.4% vs. 97.0%, *p* < 0.001). (**b**) Lymph node metastases had the strongest prognostic weight (N3, HR 14.200; N2, HR 12.945; N1, HR 3.05, all *p* < 0.001). (**c**) ALND did not confer a survival benefit on 10-year BCSS (92.1% vs. 89.5%, *p* = 0.355) in patients with 1–2 positive nodes when compared to non-ALND surgery. (**d**) Patients received ALND even had a lower 10-year BCSS than patients with non-ALND (94.7% vs. 97.1%, *p* < 0.001) in the N0 cohort.

In univariate analysis, LNM, older age (≥70), black race, poor histological type, grade 3, TNBC, and suffered multiple tumors were significantly associated with poor BCSS. These variables and ER, PR, HER2, and primary tumor site were entered into multivariate Cox regression. Results showed that LNM (N3, HR 14.200, 95%CI 7.933–25.417; N2, HR 12.945, 95%CI 7.725–21.694; N1, HR 3.05, 95%CI 2.246–4.140, all *p* < 0.001), TNBC (HR 2.825, *p* = 0.007), suffered multiple tumors (HR 2.707, *p* < 0.001), older age (≥70) (HR 2.397, *p* < 0.001), black race (HR 1.719, *p* < 0.001) and grade 3 (HR 1.375, *p* = 0.021) were poor predictors of BCSS (Table 2). Among these six predictors, LNM had the strongest prognostic weight, the N+ subgroup had a lower 10-year BCSS than the N0 subgroup (87.4% vs. 97.0%, *p* < 0.001), and the N3 subgroup had the worst 10-year BCSS with merely 49.3% (Figure 3a,b).

**Table 2:** Independent prognostic factors of breast cancer-specific survival for microinvasive breast cancer.

Characteristics	Univariate Analyses		Multivariate Analyses	
	HR (95%CI)	*p*-Value	HR (95%CI)	*p*-Value
Age (≥70 vs. 50–69)	2.561 (1.975–3.320)	<0.001	2.397 (1.840–3.123)	<0.001
Age (≤49 vs. 50–69)	1.183 (0.911–1.537)	0.207	1.159 (0.889–1.512)	0.276
Race (Black vs. White)	1.869 (1.413–2.470)	<0.001	1.719 (1.293–2.285)	<0.001
Primary site (outer/axillary tail vs. inner)	1.072 (0.761–1.511)	0.691	1.059 (0.750–1.494)	0.745
Histological type (NST vs. favorable)	1.120 (0.555–2.259)	0.752	1.020 (0.503–2.07)	0.955
Histological type (Poor vs. favorable)	3.81(1.011–14.362)	0.048	2.167 (0.557–8.431)	0.265
Grade (3 vs. 1/2)	1.461 (1.133–1.885)	0.003	1.375 (1.049–1.803)	0.021
N (1 vs. 0)	3.311 (2.457–4.463)	<0.001	3.050 (2.246–4.140)	<0.001
N (2 vs. 0)	10.879 (6.563–18.034)	<0.001	12.945 (7.725–21.694)	<0.001
N (3 vs. 0)	18.646 (10.676–32.565)	<0.001	14.200 (7.933–25.417)	<0.001
ER (positive vs. negative)	0.907 (0.708–1.163)	0.442	1.235 (0.858–1.780)	0.256
PR (positive vs. negative)	0.843 (0.668–1.065)	0.153	0.841 (0.607–1.166)	0.299
HER2 (positive vs. negative)	0.812 (0.454–1.453)	0.483	0.782 (0.338–1.808)	0.565
Sequence No. of BC (1st vs. one only)	2.376 (1.796–3.142)	<0.001	2.483 (1.870–3.298)	<0.001
Sequence No. of BC (2nd vs. one only)	2.838 (2.218–3.632)	<0.001	2.707 (2.094–3.499)	<0.001
Subtype (TNBC vs. Luminal)	2.949 (1.498–5.806)	0.002	2.825 (1.328–6.008)	0.007
Subtype (HER2 enriched vs. Luminal)	1.268 (0.609–2.638)	0.525	1.780 (0.637–4.980)	0.272

Notes: CI, confidence interval; HR, hazard ratio; No., number; NST, ductal and lobular carcinoma non-special type; TNBC, triple-negative breast cancer.

### 3.4. Survival Benefit of ALND

We further investigated the survival benefit of ALND in patients with N0 and 1–2 positive nodes, respectively. Baseline characteristics of patients between ALND and non-ALND subgroups in cohort with 1–2 positive nodes were roughly the same (Table 3). Kaplan–Meier analyses showed that ALND did not confer a survival benefit on 10-year BCSS (92.1% vs. 89.5%, *p* = 0.355) in patients with 1–2 positive nodes compared to non-ALND surgery (Figure 3c). Because significant biases existed in the N0 cohort, 1:3 PSM was used to minimize biases (Table 3). After matching, Kaplan–Meier analyses showed that patients who received ALND even had a lower 10-year BCSS than patients with non-ALND (94.7% vs. 97.1%, *p* < 0.001) in the N0 cohort (Figure 3d).

## 4. Discussion

In this study, 19.5% of patients received ALND, 80.5% were non-ALND; the LNM rate was 6.4%, including 4.3% macrometastases and 2.1% N1mi. Of note, the LNM rate reduced to 1.3% macrometastases and 1.6% N1mi in the non-ALND subgroup, slightly lower than 2% and 3% in the latest meta-analysis by Choi [6]. With a median follow-up of 96 months, MBC showed an excellent survival with a 10-year BCSS of 96.3% in the total cohort. However, 10-year BCSS declined to 87.4% in the N+ subgroup and 49.3% in the N3 subgroup. Among clinicopathological risk factors, LNM was the strongest poor prognostic predictor.

As for varied LNM rates (1.5–20%) reported in the literature [6,8,13,14,15,16], this may be partly attributable to different diagnostic criteria of MBC, inconsistent definitions of LNM, and varied SLN tracer and SLN evaluating methods. Firstly, though the concept “microinvasion” was proposed in 1982 [17], the definition of microinvasion was not internationally standardized until the 3rd edition of the WHO classification of tumors [18]. The location of tumor cells was confined to non-specialized interlobular stroma before 2012, which are likely to have deeper invasion than in the specialized interlobular stroma [19]. We did find that patients from 2003 to 2011 had a higher LNM rate in this study. Secondly, there was inconsistency in the classification of ITCs; some studies included ITCs into N+, leading to a high LNM rate [8,9,15]. Considering that some ITCs may be an iatrogenic transit of tumor cells to lymph nodes, not actual metastases, we classified ITCs into N0 based on AJCC staging [12]. Furthermore, research demonstrated that fluorescence imaging with indocyanine green is superior to a single technique with blue dye or radioisotope for SLN identification [20]. Additionally, immunohistochemistry staining can detect more N1mi and ITCs [13,21]. Beyond that, patients with ALND were not included in those studies.

As for the axillary staging of MBC, some studies suggested that MBC had very similar survival rates to DCIS, and it should be treated and followed up as pure DCIS [2,13]. Others suggested that axillary staging may be an unnecessary intervention that does not change MBC management for the majority of MBC diagnosed postoperatively [6], and even routine SLNB is perhaps not useful for MBC [8]. However, 10-year BCSS declined from 97.0% in the N0 subgroup to 87.4% in the N+ subgroup in this study, which was in line with the results of three other research based on the SEER dataset [4,5,10]. These large-scale studies compared clinicopathological features, treatments, and outcomes of pure DCIS, MBC, and T1a invasive BC. They all demonstrated that MBC seemed to resemble T1a invasive BC with more aggressive characteristics such as ER− (22.9–33.1%), HER2+ (10.5–36.5%), higher LNM rate (7.6–9.6%), and worse outcomes than that of pure DCIS. Patients with MBC may have an approximately two-fold increased risk of BC-specific death compared to patients with pure DCIS [5]. However, it should be noted that Wang et al. [10] included patients diagnosed between 1990 and 2012 and restricted to the age of 20–69 years, Cosette et al. [4] included patients with Nx or node aspiration, and Sopik et al. [5] excluded patients with LNM. Thus, there were patient biases as the target population differed across the studies due to distinct research purposes.

In addition, early breast cancer has been suggested to be a systemic disease, and sometimes breast tumors metastasize with unknown primary [22,23]. For instance, 28 excluded patients in this series presented with bone, lung, or brain metastases at initial diagnosis. Usually, actual risks are not from early invasive cancer but microinvasion because invasive cancer is treated with adjuvant therapies after surgery according to guidelines. Additionally, some MBC diagnosed intra-operatively may upgrade to invasive BC postoperatively. In multivariate analyses, LNM showed the strongest prognostic weight for BCSS; 10-year BCSS for the N3 subgroup was merely 49.3%. Furthermore, Brown et al. [24] and Pereira et al. [25] showed that the blood vessels in lymph nodes could serve as an exit route for tumor cells entering the systemic circulation. Therefore, assessment of lymph node status is necessary for MBC.

Currently, the treatment concept for BC is shifted from maximal tolerate therapy to minimum effective treatment. Thus, de-escalation of axillary surgery is required to avoid postoperative complications such as lymphedema, dysfunction of the shoulder joint, nerve injury, etc., without harming the accuracy of lymph node staging and local contrast [26]. The American College of Surgeons Oncology Group Z0011 [27] and International Breast Cancer Study Group 23-01 trials [28] indicated that omission of ALND for patients with 1–2 positive SLNs had not increased local recurrence or decreased survival compared with ALND. Based on these researches, guidelines recommended that ALND is not a must for patients who met the Z0011 trial criteria [7]. In this study, we found that patients with 1–2 positive nodes did not benefit more from ALND than from non-ALND, and patients who received ALND even showed a lower 10-BCSS than those who received non-ALND in the N0 subgroup. Therefore, we suggest that non-ALND such as SLNB is required and sufficient for patients with high-risk MBC undergoing mastectomy. However, the feasibility and predictive ability of SLNB have yet to be standardized as the medical resource is unevenly distributed in the real world. A multicenter, retrospective study in China showed that about 43.5% of early breast cancer underwent SLNB, and 83.0% of patients out of one or two positive SLNs received ALND [29].

In contrast to previous single-center studies, our study is based on a national dataset with large sample size and relatively long follow-up. However, our study has several limitations due to its retrospective nature. Firstly, it may hold a risk of misclassification and missing data. A large proportion of the patients have a missing grade (33.1%), ER (9.9%), PR (12.2%), and HER2 (36.0%) status. Apart from the abovementioned features, other features such as Ki67, several foci, lymphovascular invasion, extensive in situ components, and surgical margin status are not available. Secondly, the diagnostic criteria of MBC and all the other pathological features were continually revised during a quite large timespan of the data. These, plus varied categorization of variables in studies, have significant implications in identifying prognostic factors for MBC. Thirdly, limited information is available on surgery, chemotherapy, and radiotherapy. The treatment status of patients recorded as no/unknown for chemotherapy is 91.4%, and unknown for radiotherapy is 55.7%. Though more patients were recorded as radiotherapy-received in the non-ALND subgroup than in the ALND subgroup (33.8% vs. 29.7%) during PSM analysis, more than 60% of patients remain unclear whether they received radiotherapy or not. Consequently, we cannot conclude whether radiotherapy affects prognosis in the N0 subgroup. Moreover, specific regimens of medical therapies were inaccessible.

## 5. Conclusions

Our study confirmed that though MBC has a low LNM rate (6.4%) and relatively excellent prognosis for most patients, patients with LNM showed poor prognosis, and SLNB is required and sufficient for MBC. In addition, internationally standardized diagnostic criteria and categorization are the premises for a more comprehensive and in-depth understanding. Further prospectively and retrospectively research is warranted to distinguish patients with high-risk characteristics and help tailor axillary management to guide adjuvant treatment while minimizing unnecessary interventions of MBC.

## Figures and Tables

**Table 3:** Baseline characteristics between non-ALND and ALND subgroups in cohort with N0 and 1–2 positive nodes.

Characteristics	N0 Original Cohort			N0 Propensity-Matched Cohort			1–2 Positive Nodes Cohort		
	Non-ALND n (%) (n = 9141)	ALND n (%) (n = 1797)	*p*-Value	Non-ALND n (%) (n = 5391)	ALND n (%) (n = 1797)	*p*-Value	Non-ALND n (%) (n = 267)	ALND n (%) (n = 337)	*p*-Value
Age, years			<0.001			0.361			0.05
20–49	2207 (24.1) ^a^	440 (24.5) ^b^		1356 (25.2)	440 (24.5)		86 (32.2)	117 (35.7)	
50–69	5294 (57.9) ^a^	945 (52.6) ^b^		2885 (53.5)	945 (52.6)		135 (50.6)	185 (54.9)	
≥70	1640 (17.9) ^a^	412 (22.9) ^a^		1150 (21.3)	412 (22.9)		46 (17.2)	35 (10.4)	
Race			<0.001			0.758			0.406
White	7053 (77.2) ^a^	1352 (75.2) ^a^		4076 (75.6)	1352 (75.2)		190 (71.2)	248 (73.6)	
Black	924 (10.1) ^a^	241 (13.4) ^b^		688 (12.8)	241 (13.4)		55 (20.6)	56 (16.6)	
Other ^①^/unknown	1164 (12.7) ^a^	204 (11.4) ^a^		627 (11.6)	204 (11.4)		22 (8.2)	33 (9.8)	
Histological type			0.151			0.387			0.428
Favorable type ^②^	257 (2.8)	47 (2.6)		144 (2.7)	47 (2.6)		3 (1.1)	6 (1.8)	
NST and others ^③^	8698 (95.2)	1716 (95.5)		5138 (95.3)	1716 (95.5)		258 (96.6)	323 (95.8)	
Poor type ^④^	22 (0.2)	9 (0.50)		15 (0.3)	9 (0.5)		1 (0.4)	0 (0.0)	
Unspecified	164 (1.8)	25 (1.4)		94 (1.7)	25 (1.4)		5 (1.9)	8 (2.4)	
Grade			0.004			0.403			0.059
1	1549 (16.9) ^a^	285 (15.9) ^a^		862 (16.0)	285 (15.9)		38 (14.2)	44 (13.0)	
2	2462 (26.9) ^a^	436 (24.3) ^b^		1405 (26.0)	436 (24.3)		59 (22.1)	105 (31.2)	
3	2088 (22.8) ^a^	475 (26.4) ^b^		1347 (25.0)	475 (26.4)		76 (28.5)	95 (28.2)	
Unknown	3042 (33.3) ^a^	601 (33.4) ^a^		1777 (33.0)	601 (33.4)		94 (35.2)	93 (27.6)	
ER			<0.001			0.002			0.156
Negative	2264 (24.8) ^a^	494 (27.5) ^b^		1394 (25.9) ^a^	494 (27.5) ^a^		174 (65.2)	241 (71.5)	
Positive	6055 (66.2) ^a^	1027 (57.2) ^b^		3313 (61.4) ^a^	1027 (57.2) ^b^		27 (10.1)	22 (6.5)	
Borderline/unknown	822 (9.0) ^a^	276 (15.3) ^a^		684 (12.7) ^a^	276 (15.3) ^b^		66 (24.7)	74 (22.0)	
PR			<0.001			0.005			0.085
Negative	3310 (36.2) ^a^	668 (37.2) ^a^		1953 (36.2) ^a^	668 (37.2) ^a^		92 (34.5)	118 (35.0)	
Positive	4791 (52.4) ^a^	810 (45.1) ^b^		2629 (48.8) ^a^	810 (45.1) ^b^		141 (52.8)	194 (57.6)	
Borderline/unknown	1040 (11.4) ^a^	319 (17.7) ^b^		809 (15.0) ^a^	319 (17.7) ^b^		34 (12.7)	25 (7.4)	
HER2 (from 2010)			<0.001			0.192			0.002
Negative	2044 (42.3) ^a^	232 (41.2) ^b^		783 (42.7)	232 (41.1)		52 (41.0) ^a^	77 (56.6) ^a^	
Positive	1003 (20.7) ^a^	126 (22.3) ^b^		387 (21.1)	126 (22.4)		39 (30.7) ^a^	42 (30.9) ^a^	
Borderline/unknown	1788 (37.0) ^a^	206 (36.5) ^b^		663 (36.2)	206 (36.5)		36 (28.3) ^a^	17 (12.5) ^b^	
Molecular subtype (from 2010)			<0.001			0.373			<0.001
Luminal	2315 (47.9) ^a^	261 (46.2) ^b^		878 (47.9)	261 (46.2)		65 (51.2) ^a^	88 (64.7) ^a^	
Triple-negative	246 (5.1) ^a^	27 (4.8) ^b^		76 (4.1)	27 (4.8)		9 (7.1) ^a^	12 (8.8) ^a^	
HER2 enriched-type	468 (9.7) ^a^	67 (11.9) ^b^		208 (11.4)	67 (11.9)		16 (12.6) ^a^	19 (14.0) ^a^	
Unknown	1806 (37.3) ^a^	209 (37.1) ^b^		671 (36.6)	209 (37.1)		37 (29.1) ^a^	17 (12.5) ^b^	
Primary site			<0.001			0.322			0.544
Inner	1558 (17.0) ^a^	226 (12.6) ^b^		752 (14.0)	226 (12.6)		36 (13.5)	38 (11.3)	
Outer/Axillary tail	3880 (42.5) ^a^	769 (42.8) ^a^		2293 (42.5)	769 (42.8)		90 (33.7)	126 (37.4)	
Other	3703 (40.5) ^a^	802 (44.6) ^b^		2346 (43.5)	802 (44.6)		141 (52.8)	173 (51.3)	
Sequence No. of breast cancer			0.576			0.894			0.204
One primary only	6320 (69.1)	1220 (67.9)		3661 (67.9)	1220 (67.9)		172 (64.4)	240 (71.2)	
1st of multi-tumors	1151 (12.6)	234 (13.0)		721 (13.4)	234 (13.0)		42 (15.7)	43 (12.9)	
≥2nd of multi-tumors	1670 (18.3)	343 (19.1)		1009 (18.7)	343 (19.1)		53 (19.9)	54 (15.9)	
Primary site surgery			0.513			1			0.052
No/unknown	6 (0.07)	2 (0.11)		5 (0.1)	2 (0.1)		5 (1.9)	1 (0.3)	
Yes	9135 (99.9)	1795 (99.9)		5386 (99.9)	1795 (99.9)		262 (98.1)	336 (99.7)	
Chemotherapy			<0.001			0.152			<0.001
No/unknown	403 (4.4)	131 (7.3)		339 (6.3)	131 (7.3)		117 (43.8)	234 (69.4)	
Yes	8738 (95.6)	16,686 (92.7)		5052 (93.7)	1666 (92.7)		150 (56.2)	103 (30.6)	
Radiotherapy			<0.001			0.004			0.236
Yes	4307 (47.1) ^a^	533 (29.7) ^b^		1824 (33.8)	533 (29.7)		83 (31.1)	95 (28.2)	
No/unknown	4764 (52.1) ^a^	1256 (69.9) ^b^		3550 (65.9)	1256 (69.9)		184 (68.9)	239 (70.9)	
Refused	70 (0.8) ^a^	8 (0.4) ^a^		17 (0.3)	8 (0.4)		0 (0.00)	3 (0.90)	

Notes: ① Other races are Asian or Pacific Islander and American Indian/Alaska Native. ② Favorable type include adenoid cystic, tubular, encapsulated papillary, mucinous, and cribriform carcinoma. ③ NST and others include ductal and lobular carcinoma non-special type (NST), intracystic, medullary, and apocrine carcinoma. ④ Poor type includes micropapillary, clear cell, signet ring cell, and metaplastic carcinoma. a, b Different marks a and b indicate that pairwise comparisons are statistically significant between subgroups. The same marks indicate not statistically significant, such as a and a. ALND, axillary lymph node dissection; CI, confidence interval; No., number; OR, odds ratio; SLNB, sentinel lymph node biopsy.

## Data Availability

The datasets used and analyzed during the current study are available from the corresponding author on reasonable request.
